# Segawa Syndrome, a Dramatic Response to Dopamine

**DOI:** 10.1155/2024/8154006

**Published:** 2024-03-31

**Authors:** Omkar Dhungel, Amit Shrestha, Pawan Sharma, Nidesh Sapkota, Raju Paudel

**Affiliations:** ^1^Department of Psychiatry, Patan Academy of Health Sciences, Lagankhel, Lalitpur, Nepal; ^2^Department of Neurology, Grande International Hospital, Tokha, Kathmandu, Nepal

## Abstract

Segawa syndrome usually manifests as dystonia, disturbance of gait with fatigue, and may be confused with spasticity. Also known as dopamine-responsive dystonia (DRD), it should be considered in any child who presents with paroxysmal or progressive hypertonia of unknown etiology, which responds dramatically to levodopa. It is a clinical diagnosis, but the level of pterins in cerebrospinal fluid and guanosine triphosphate cyclohydrolase-1 (GTCH 1) gene mutation testing done by molecular genetic testing are confirmatory. Our case is a 45-year female with a family history of similar illness expressed as autosomal recessive inheritance pattern. She had symptoms onset at an early age of 13 years with features of dystonia of predominantly lower limbs, hence the inability to maintain posture and walk. Dramatic improvement with levodopa but sudden deterioration to dystonia due to noncompliance was evident in our patient with troublesome features of concomitant adjustment disorder during presentation.

## 1. Introduction

Segawa syndrome, also known as dopamine-responsive dystonia, is rare hereditary progressive dystonia with marked diurnal fluctuation, with an estimated prevalence of 0.5 per million [1]. The two types of hereditary DRD due to dopamine deficiency in the nigro-striatum dopaminergic neurons have been described. Among them, the autosomal dominant pattern was originally described by Segawa as hereditary progressive dystonia with marked diurnal fluctuation. It is caused by an enzymatic defect of GTCH 1 and autosomal recessive pattern is caused by enzymatic defect of tyrosine hydroxylase (TH), both of which will ultimately lead to dopamine deficiency in nigro-striatal terminal. Hence, the motor dysfunction of the syndrome occurs. Disease caused by a recessive mutation in the TH gene is rare and severe [2]. The motor symptoms of Segawa syndrome usually become apparent by around six years of age and intelligence is not affected. Women are affected four times more often than men and are also more likely to have severe symptoms than men [3].

We present a case of an autosomal recessive pattern of inherited dopamine-responsive dystonia with adjustment disorder in a 45-year-old female in a resource-limited setting, where we relied on our clinical acumen rather than on genetic analysis or specific imaging modalities.

## 2. Case History

**Figure 1:**
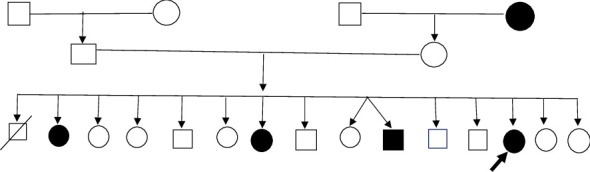
Family tree of our patient, showing the presence of similar symptoms of dystonia in other members of the family.

A 45-year-old unmarried female presented with an illness of 32 years duration, which started when she was 13 years of age. There was strong genetic loading with similar symptoms in 1^st^ and 2^nd^ degree relatives. 2 elder sisters and 1 elder brother and maternal grandmother (Figure 1) had varying nature of symptomatic manifestation from mild gait disturbances to weakness of all 4 limbs and inability to hold body in appropriate posture and were bed ridden. All of them had an early age of onset in their teens. There was no known psychiatric illness in the family and no history of any chronic medical illness.

The onset was subacute with gradual progression of generalized weakness which was followed by inability to move all the extremities and plantar flexion and inversion of the ankle with tightness and posturing. She would not be able to go to the toilet by herself. For this ailment, she was hospitalized for 5 days and started on tablet levodopa from the age of 14 years, for the past 31 years. She was maintaining well with 275 mg of levodopa and if she missed a single dose, there would be exacerbation of symptoms.

**Figure 2:**
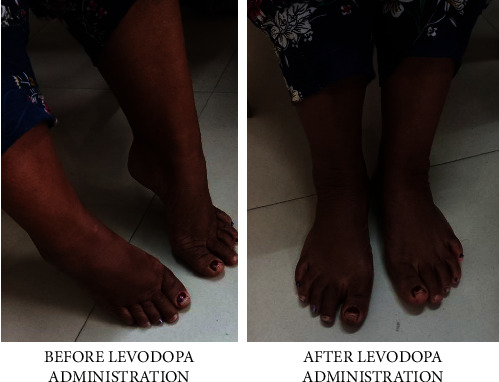
Posture of the foot before and after levodopa.

However, in December 2022, she presented with complaints of burning sensation, increased sweating, and inability to move for around 2 months as she was noncompliant with medication due to financial issues. She developed a prominent spasticity of the dominant bulk of muscles of the lower limb. There was plantar flexion and inversion of bilateral ankle joint with dorsiflexion of meta-tarso-phalangeal joint and flexion of inter-phalangeal joints (Figure 2). This ailment was associated with a feeling of low mood (not persistent and pervasive) for about 1 month and hopelessness as she felt that the illness would make her paralyzed. She also had death wishes, deranged sleep cycle, restlessness, helplessness, decreased self-esteem, and decreased appetite, with a burning sensation so severe that she would not be able to put on clothes, on the days when she refrained from taking medicine. For this she self-medicated with tablet levodopa on some days which would improve the spasticity, but her mood symptoms were persisting.

On neurological assessment, higher mental function, cranial nerves, and sensory function were normal. Motor examination revealed normal muscle bulk but increased tone in all 4 limbs. The power in the lower limbs was 4+ with brisk knee reflex and plantar flexion bilaterally. However, there were no signs of meningeal irritation, cerebellar dysfunction, or autonomic dysfunction. On mental state examination, there was increased reaction time during interview, sad affect, decreased self-confidence, and death wishes. Beck Depression Inventory score was 19 (Borderline clinical depression). The baseline investigations included complete blood count, liver function test, renal function test, electrolytes, random sugar, thyroid function test, and MRI brain which were normal.

A combined neurological and psychiatric evaluation came up with the provisional diagnosis of Segawa disease with adjustment disorder (according to International Classification of Diseases 11th Revision) and the patient was started on with levodopa + carbidopa (100 + 25 mg) which was optimized in follow-up and Cognitive Behavioral Therapy was started. Following the integrated therapy, there has been significant improvement in both neurological and psychiatric symptoms, and she is maintaining well till 6 months of follow-up.

## 3. Discussion

In Autosomal Dominant DRD, a specific alteration in the GTCH 1 gene located on chromosome 14q results in reduced GTCH 1 activity, leading to decreased levels of tetrahydrobiopterin, TH, and dopamine in the nigro-striatal terminal. On the other hand, Autosomal Recessive DRD is characterized by various mutations in the TH gene (located on chromosome 11p15.5), resulting in diminished tyrosine hydroxylase enzyme activity [4]. This enzyme is crucial for synthesizing catecholamines such as dopamine, epinephrine, and norepinephrine, which play vital roles in regulating motor coordination, behavior, learning and memory, the sleep-wake cycle, endocrine processes, and visceral function. Hence, motor, mood, and cognitive symptoms are prevalent in this condition [5].

Segawa syndrome typically presents between infancy and adolescence with an average age of onset of 8 to 11 years. In our patient, the disease has been 1^st^ diagnosed during her early teens, i.e., 13 years of age. However, in males, disease onset is late with the milder phenotype and the penetrance of mutation is lower than that in females [6]. Dystonia typically starts in the leg as an action dystonia that leads to equinovarus foot posturing, hence impairing the overall motor movements. This typical clinical presentation is apparent in our case too as depicted in the picture (Figure 2). Diurnal fluctuation is common with dystonia worsening towards the end of the day. The degree of diurnal fluctuation diminishes with age with little or no fluctuation remaining by the third decade [6], which is also the presentation in our patient in her 40s.

Atypical clinical features of Segawa syndrome include oculogyric crises, waddling gait, generalized hypotonia, proximal weakness, brisk tendon reflexes, ankle clonus, Parkinsonism-like features of rigidity, bradykinesia, and postural tremors, especially common when symptom onset is after 15 years of age [7]. In atypical presentations, there might be partial improvement with levodopa and also an associated dose limiting side effect of levodopa, i.e., dyskinesias. The dyskinesias should not refrain the clinician from using levodopa trials in a case of dystonia [8]. The atypical symptoms were not prominent in our case.

DRD diagnosis primarily depends on clinical signs and response to levodopa/carbidopa treatment. For confirmation, tests like phenylalanine loading and CSF analysis for biogenic amines, pterins, and GTCH 1 enzyme activity are conducted. Despite being invasive, CSF analysis remains the most reliable diagnostic method. DRD includes cases without gene mutations but responding to levodopa. Initiating a levodopa trial is recommended for diagnosis, especially in resource-limited settings, like ours, where clinical presentation guides diagnosis [9, 10].

Meanwhile, so far, nonmotor symptoms have been overlooked and very few and often contrasting data are currently available on the matter. Mood disturbances (depression, anxiety, OCD, and impulsivity), deranged sleep (increase of daytime sleepiness along with the impairment of nocturnal sleep structure), and cognitive impairment are reported in scattered literature [10]. In our patient, the inability to afford medication and worsening symptoms due to noncompliance resulted in adjustment disorder with depressive features. These included death wishes, disrupted sleep, restlessness, feelings of helplessness, reduced self-esteem, decreased appetite, and widespread burning hyperesthesia. Additionally, mobility and functional issues exceeded her ability to cope. While withdrawal of dopamine medication might have contributed to depressive symptoms, psychiatric manifestations persisted even after resuming levodopa.

When considering diagnoses like cerebral palsy and juvenile Parkinson disease (JPD), certain factors distinguish them. For instance, the absence of intellectual disability, positive response to dopamine therapy, and lack of convulsions despite increased muscle tone and spasms exclude cerebral palsy [11]. JPD requires long-term levodopa intake and can lead to motor fluctuations and dyskinesias, and DRD shows significant improvement with dopamine therapy. Imaging techniques like DAT (Dopamine Transporter) imaging or fluorodopa PET (Positron Emission Tomography) help differentiate between them. DAT imaging reveals decreased binding in JPD cases but remains normal in DRD, while fluorodopa PET scans show reduced uptake in JPD patients but normal uptake in those with DRD [11].

The limitation of our study was that we had to rely on clinical acumen and could not do the necessary investigations to confirm the diagnosis of Segawa disease in our resource-limited setting. Our experience further underscores the broad clinical presentations of DRD and advocates for the diagnostic value of trying levodopa and keeping genetic testing in dystonia for research purposes.
